# The Expression of GPR30 in Iron-Overloaded Atypical Ovarian Epithelium and Ectopic Endometrium Is Closely Correlated with Transferrin Receptor and Pi3K

**DOI:** 10.1155/2022/8338874

**Published:** 2022-09-12

**Authors:** Li Long, Zhaoning Duan

**Affiliations:** ^1^Department of Health Management Center, The First Affiliated Hospital of Chongqing Medical University, China; ^2^Department of Gynecology, The First Affiliated Hospital of Chongqing Medical University, China

## Abstract

**Background:**

The mechanism of atypical hyperplasia of the ovarian epithelium and ectopic endometrium caused by iron overload remains unclear. Accordingly, we investigated possible effects on the human ovarian ectopic endometrium and ovarian epithelium by producing a high-iron environment with rat ovaries.

**Objective:**

Human ovarian ectopic endometrium with atypical hyperplasia was collected, and the correlation between transferrin receptor GPR30 and Pi3K protein expression was studied by immunohistochemistry staining. Twenty SPF Sprague–Dawley female rats were microinjected with iron into one side of the ovary once a month, and the other ovary was used as the control. After 10 months of microinjection, the iron histological analysis was examined by Prussian blue staining, and ovarian endometrium morphology was assessed by HE staining. Abnormal lesion changes were measured by Pi3K staining. Evaluation of GPR30 was performed using reverse transcription PCR (RT-PCR) and western blotting, and the interrelationship between GPR30 and Pi3K was also assayed.

**Results:**

GPR30 was significantly increased and correlated with the transferrin receptor and Pi3K in atypical human ovarian ectopic endometrium. Iron overload was confirmed in the 20 microinjected ovary cortexes, epithelial hyperplasia was observed in 12 ovaries, and papillary atypical hyperplasia was noted in eight ovaries. The RNA and protein levels of GPR30 were significantly increased in atypical hyperplasia compared to hyperplasia tissue samples. A positive relationship between GPR30 and Pi3K was found (*P* = 0.001).

**Conclusion:**

The results suggest that persistent iron exposure may be a potential stimulus for ovarian endometriosis with atypical changes, and the abnormal increase in the new estrogen receptor GPR30 is closely related to this process.

## 1. Introduction

Endometriosis is a very common gynecologic disorder in reproductive-age women that is defined as the implantation of endometrium-like glandular and stromal cells outside the uterus. Although benign in nature and clinical behavior, endometriosis is associated with an increased risk of malignant transformation. Molecular and epidemiological studies have also provided a strong association between atypical endometriosis and ovarian cancer [1, 2]. It has been reported that atypical endometriosis occurs in up to 6.8% of ovarian cancer cases, and ovarian endometrioid carcinoma originating from ectopic endometrium is more prone to atypical changes [3]. These factors, therefore, demonstrate a high risk of type I ovarian cancer in women with these atypical cells and/or the structure of ovarian endometriosis. Considering the obstacles to early detection (screening) of ovarian cancer and the remarkable results of treatment, but with relatively limited success, it is worth studying the hyperplasia and especially the atypical changes of endometriosis to shed light on new perspectives on ovarian cancer.

Endometriomas, also called chocolate cysts or endometrioid cysts, repeatedly bleed during the menstrual cycle and are gradually filled with thick, viscous, and decomposed blood that is often referred to as “old blood,” which appears dark, brownish red, or chocolate-like. After decomposition, the old blood forms and accumulates a large number of iron metabolites. Iron is the most abundant heavy metal in mammals and is an essential element and critical component of the molecules involved in energy production, the cell cycle, and intermediate metabolism. However, excess of iron results in toxicity and is linked to pathological disorders. Nonprotein-bound “free” or “catalytic” iron functions to damage biomolecules with a hypothetical “site-specific” mechanism [4]. Additional evidence also suggests that the role of local iron overload is a major pathology associated with carcinogenesis [5]. In endometriosis, iron overload has been demonstrated to reveal persistently high affinity with ovarian endometriosis-associated stromal cells [6]. However, the major pathologies and possible underlying mechanisms between iron metabolites and the local ovarian epithelium are still not sufficiently studied.

In recent years, GPR30, a 7-transmembrane G protein-coupled receptor family member, has been reported to be related to rapid and transcription-mediated responses to estrogen in certain circumstances [7]. Furthermore, clinical studies have shown that GPR30 overexpression is associated with the incidence of patients with poor prognosis of ovarian and endometrial cancer [8, 9], which suggests that GPR30 may play an important role in carcinogenesis. Our previous study also found that abnormally high expression of GPR30 may be involved in endometriosis-associated ovarian carcinoma [10]. In the present study, we first hypothesized that GPR30, a novel ER, may be involved in the major pathologies of the atypical changes, which induced by iron overload of ovarian ectopic endometrium and persistent microinjection of iron into the rat ovarian intraepithelium.

## 2. Materials and Methods

### 2.1. Samples

**Figure 1:**
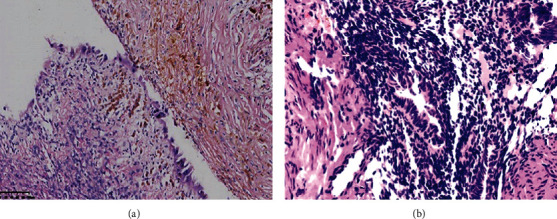
HE Staining of Atypical Hyperplasia Human Ovarian Endometrium. (a): Ovarian ectopic endometrium. (b): Atypical ovarian ectopic endometrium.

A total of 20 atypical ectopic endometrial samples were obtained from the Pathological Diagnosis Center of Chongqing Medical University, and this study was approved by the ethics committee of Chongqing Medical University. The inclusion of atypical endometriosis cases was performed by two pathologists with a double evaluation (Figure 1). In addition, 20 samples of normal endometriosis were collected as a control. Informed consent of the participants was obtained for all samples.

### 2.2. Animals

All animal experiments were conducted in accordance with the approval and guidelines of the Animal Care and Use Committee at Chongqing Medical University. All strategies were made to minimize the number of animals used and their suffering.

Twenty SPF Sprague–Dawley female rats that were two months of age (weighing 160 g to 180 g) were provided by the animal center of Chongqing Medical University (purchased from Nanjing Institute of Animal Research) and housed in a 12-hour light/dark cycle under controlled temperature and humidity with free access to food and water.

### 2.3. Rat Ovary Iron Overload Model by Microinjection

**Figure 2:**
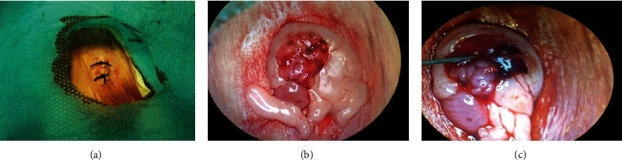
Rats were subjected to a small incision at the right back body surface (a) of the ovary (b). Iron dextran for injection was microinjected into the subenvelope of the ovary (c).

All experimental rats were fasted overnight but were allowed free access to water. The body weight of each rat was calculated prior to surgery. Anesthesia was induced with pentobarbital sodium at a dose of 150 mg/kg by intraperitoneal injection. Rats were placed in the prone position, and a small incision of approximately 1.0 cm was made on the surface of the right posterior body of the ovary. The derma, muscular layer, and peritoneum were cut in sequence, and the right ovary was removed from the abdominal cavity. Then, 40 *μ*l (1 mg Fe equivalent) of iron dextran for injection was microinjected into the subenvelope of the ovary and superficial ovarian tissue. The cut was then sutured with a 4/0 nylon thread. The other side, the left-side ovary, was the control, and glucose water was microinjected (Figure 2). The second microinjection was performed 30 days after the first operation. The animals were killed 10 months after microinjection.

### 2.4. Staining of Iron Deposits

Ferric iron deposits were detected in ovarian tissue in the two groups (control and iron) using Prussian blue staining. Paraffin sections were dewaxed, rehydrated with water, stained with liquid Perl's solution for 20–30 min, thoroughly washed in distilled water after 0.5% basic fuchsin solution for 30–50 s, and followed by rapid differentiation with 95% alcohol, dehydration with anhydrous alcohol and clearing with xylene. Sections were observed under an inverted optical microscope after neutral gum cementing.

### 2.5. Morphology

Twenty ovaries (iron microinjected) were necropsied, put into 4% buffered paraformaldehyde, and embedded in paraffin. Then, 10 *μ*m thick serial sections were stained with hematoxylin and eosin (HE stains) for histological evaluation. Next, phosphoinositide 3-kinase (Pi3K) immunohistochemical (IHC) staining was carried out. Briefly, after inhibition of endogenous tissue peroxidase, unmasking of the antigen and inhibition of nonspecific reactions in a blocking solution containing 10% normal goat serum and 1% bovine serum albumin were conducted, and sections were kept overnight in a 1/500 dilution of monoclonal Pi3K antibody (sc-56935, Santa Cruz Biotechnology, USA 1 : 500, purchased from Shengbo Reagent Co). Staining was performed with peroxidase-labeled secondary antibody and liquid diaminobenzidine as the chromogen. The twenty control ovaries were treated in the same way.

### 2.6. Immunohistochemical Staining and Analysis

As above, immunohistochemical staining at 1 : 200 for GPR30 (sc-48525) and transferrin receptor (TfR; sc-32272) was performed using avidin-biotin-peroxidase methods. The entire tissue section was evaluated by objective Hscore to measure the staining intensity, and the percentage of stained tumor cells was positive. Five fields of view of each section were randomly selected, the magnification was 400×, and the number of positive cells was counted per 100 cells per field. Staining intensity was classified as negative (<5% positive tumor cells), 1+ (mild), 2+ (medium intensity), or 3+ (greater than the intensity of the positive control). Two pathologists at the Pathological Diagnosis Center of Chongqing Medical University used double-blind methods to evaluate the sections to determine the immunohistochemical results. The formula was Hscore = *Σ* (*i* + 1) × pi, where *i* represents the intensity of staining, pi is the percentage of positive cells stained/total number of test cells, and the correction factor is 1.

### 2.7. Reverse Transcription Polymerase Chain Reaction (RT-PCR) Analysis

Total RNA was extracted from microdissected ovarian epithelium (including atypical papillary hyperplastic epithelium, hyperplastic epithelium, and control ovary epithelium) stored at -80°C using the RNAiso plus (D9108A, Takara, Japan) extraction method, as described in the manufacturer's directions. Briefly, the total RNA was extracted with RNAiso plus, precipitated with isopropanol, washed in ethanol, and resuspended in RNase-free water. The quantity and quality of RNA were determined by spectrophotometry. Two micrograms of total RNA was used for reverse transcription (RT) with RT kits (DRR037A, Takara, Japan) as described in the manufacturer's directions. The specific primers for GPR30 mRNA were F1 5′-CACCCCTCCGCCTGGAGAGC-3′, R1 5′-CACAGTCCCGCGTGGAGC-3′, and the product size was 346 bp; the specific primers for the beta-actin gene were F1 5′-CTCGTCATACTCCTGCTTGCTG-3′, R1 5′-CGGGACCTGACTGACTACCTC-3′, and the product size was 546 bp. For amplification of both GPR30 and the reference gene beta-actin, the following PCR protocol was applied: GPR30: 95°C, 5 min; 95°C, 30 s; 56°C, 30 s; 72°C, 30 s; 72°C, 10 min; *β*-actin, 95°C, 5 min; 95 and 59.5°C, 30 s; 72°C, 30 s; and 72°C, 10 min.

### 2.8. Western Blotting Analysis

Total protein extracts from the ovary tissues (the samples were the same as RT-PCR) were stored at -80°C, and equal amounts of the protein obtained using lysis buffer were analyzed by immunoblotting (30 *μ*g per sample). Anti-*β*-actin antibody was obtained from Sigma-Aldrich (A5441, 1: 2000). The antibody used was directed against rabbit anti-human GPR30 IgG (sc-48525; Santa Cruz Biotechnology, USA, 1: 1000). The secondary antibody was an anti-rabbit immunoglobulin horseradish peroxidase-linked F (ab) 2 fragment (Amersham Biosciences) from donkey. Western blotting reagents were obtained from an electrochemiluminescence kit (Amersham Biosciences).

### 2.9. Statistical Analysis

The outcome of IHC staining and RT-PCR was carried out by semiquantitative analysis with Image-Pro Plus 6.0 and SPSS 16.0 software. Data are expressed as the mean ± SEM. To compare means between the two groups, we used an independent-samples *T* test, and the *P* < 0.05 level was considered significant. The association between the expression of biomarkers (i.e., GPR30 and Pi3K) was analyzed with contingency tables.

## 3. Result

### 3.1. GPR30 And TfR Upregulation in Human Atypical Ectopic Endometrium

**Figure 3:**
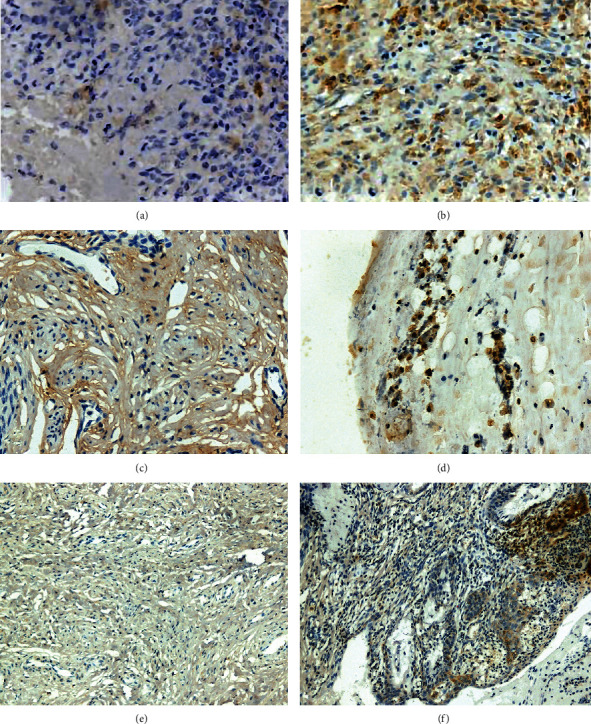
GPR30, iron metabolites TfR and Pi3K protein were more positively expressed in the atypical human ovarian endometrium (b, d, f) than in the normal control group (a, c, e).

The GPR30 and TfR were more positively expressed in the human atypical ectopic endometrium group than those in the control group. The antibody effectors of GPR30, TfR, and Pi3K were brown-yellow particles located in the cytoplasm and membrane (Figure 3). GPR30 was positively expressed in 20 cases (100%) of atypical ectopic endometrium, and the average semiquantitative HScore was 305 (0–356); moreover, GPR30 was positively expressed in 3 cases (15%) of benign ectopic endometrium, and the average HScore was 62 (0–180). The positive rate of TfR products in atypical ectopic endometrium and the HScore value were also higher than the expression level of control endometrium (*P* <0.05) (Table 1).

### 3.2. GPR30 Correlated Significantly with TfR and Pi3K in Human Atypical Ectopic Endometrium

For the atypical ectopic endometrium, the HScore analysis suggested that the expression levels of GPR30 and Pi3K were both significantly increased. The pairwise correlation between the expression of GPR30 and iron metabolites and Pi3K was examined, and the expression of GPR30 significantly correlated with TfR and Pi3K immunopositivity in the atypical ectopic endometrium group (*P* = 0.001, Tables 2).

### 3.3. Iron-Induced Histological Changes in Rat Ovary

**Figure 4:**
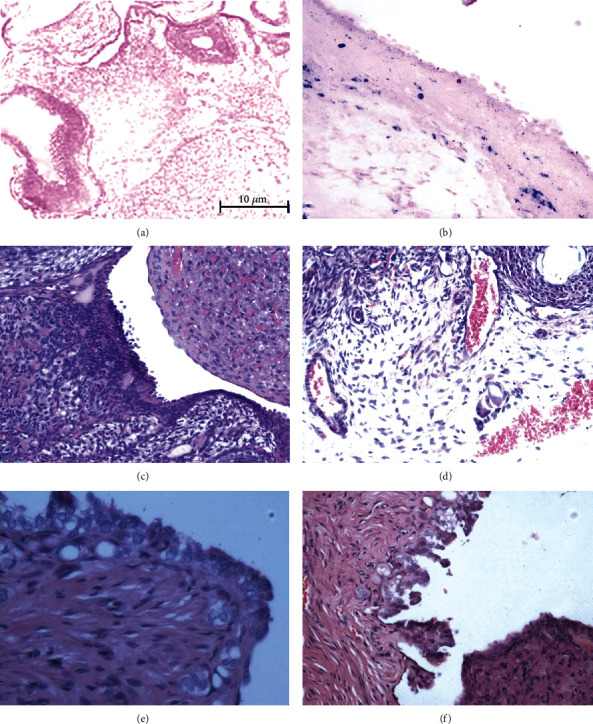
Ferric iron (blue) deposits of ovaries with Prussian blue staining in the endometriosis model group (b) by repeated injections of iron, with most of the iron (Fe^+2^) accumulated in macrophages. There were no ferric iron deposits identified in control ovary tissue (a). Repeated microinjections of iron led to different morphologic changes in the ovarian epithelium: multinucleated giant cells (d), epithelial hyperplasia (e), and papillary hyperplasia with atypical cells (f) in iron-dextrin-injected ovaries. Image (c) is control ovary tissue injected with glucose water. No cellular morphology changes were observed in the control ovary tissues, which clearly indicate that the tissue reaction observed was not induced by surgical trauma or dextrin as an iron carrier substance.

Ferric iron deposits in ovaries were evidenced using Prussian blue staining. Before injection, the control ovarian tissues were devoid of iron deposits. In injected ovarian tissues, as illustrated, the deposits were particularly numerous in the interface area between the ovarian tissues and the ovarian epithelium (Figure 4). Iron deposits were never observed in the control group.

Chronic inflammation reaction performance, macrophages, and multinucleated giant cells were seen in all 20 ovaries in the iron overload model, epithelial hyperplasia was seen in 12 ovaries, and papillary hyperplasia with atypical cells was seen in the serosal cells of 8 ovaries (Figure 4). None of these pathologic changes were observed in the control ovary tissues, and normal endometriosis did not feature papillary hyperplasia with atypical cells.

### 3.4. Upregulation of GPR30 in Iron-Induced Atypical Hyperplasia

**Figure 5:**
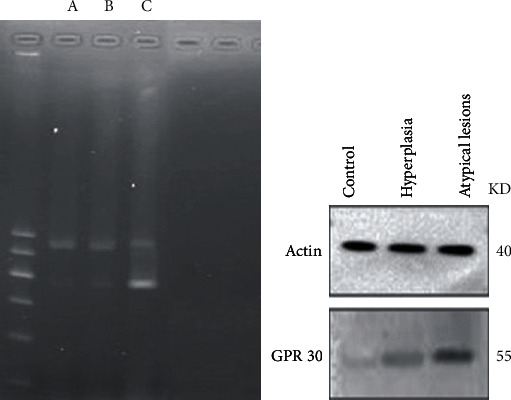
GPR30 mRNA (346 bp) and protein were significantly more expressed in the atypical group (c) than in the epithelial hyperplasia (b) and control groups (a). These data suggest that repeated microinjection of iron may have an effect on the expression of GPR30, with increased expression of GPR30 most obvious in atypical alterations of the ovarian epithelium.

The final gel and protein electrophoresis pictures were analyzed with Quantity One®1-D analysis software (Bio-Rad). The relative expression levels of GPR30 mRNA and protein in the atypical group were 0.831 ± 0.003 and 0.838 ± 0.001, respectively; those in the hyperplasia group were 0.132 ± 0.025 and 0.127 ± 0.003, respectively; and those in the control group were 0.056 ± 0.012 and 0.047 ± 0.003, respectively, (*P* < 0.05, Table 3). The GPR30 mRNA and protein expression levels were significantly higher in the atypical group than in the control group (Figure 5), suggesting that there was transcriptional activation of GPR30 gene expression in atypical hyperplasia, which led to a marked increase in the secreted expression of GPR30, as detected by western blotting.

### 3.5. GPR30 Correlated Significantly with Pi3K in Atypical Hyperplasia

**Figure 6:**
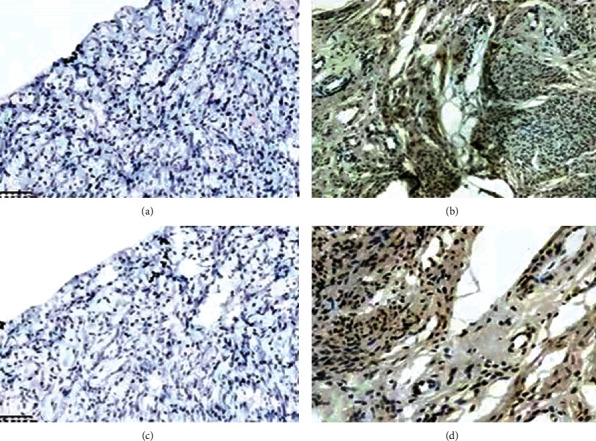
Pi3K and GPR30 proteins (brown granules located in the nucleus and cytoplasm) were significantly increased in expression in the ovarian epithelial cells of the atypical group (b, d) than in the control group (a, c). This finding suggests that the occurrence of atypical ovarian epithelia by repeated microinjection of iron can lead to the upregulation of GPR30 and Pi3K. Images are shown at 400× magnification.

For IHC staining, Pi3K, and GPR30 proteins were more positively expressed in the ovarian epithelial cells of the model group than in the control group (Figure 6). HScore analysis suggested that the expression levels of Pi3K and GPR30 were both significantly increased in the atypical hyperplasia group (323/0–358; 309/0–325) compared with the hyperplasia group (12/0–110; 25/0–180) (*P* < 0.05, Table 4). The pairwise correlation between the expression of GPR30 and Pi3K was examined. The expression of GPR30 significantly correlated with Pi3K immunopositivity in the atypical hyperplasia group (*P* = 0.001, Table 5), which occurred more often in atypical hyperplasia cells whose results was consistent with their expression in atypical epithelial changes of human ectopic endometrium.

## 4. Discussion

On the basis of the histological findings, it is possible to stratify the impact of iron overload on human tissues into significantly different groups. On one hand, iron is an essential element for physiological functions, such as hematopoiesis and immunology. On the other hand, excessive iron deposition can affect the physiological function of cells, leading to pathological changes and even malignant transformation [11–13]. Studies have shown that persistent abnormal iron uptake in organs leads to hyperplasia, adhesion, DNA damage derived from free radical toxicity, and chronic inflammation in organs such as the liver [14, 15], breasts [16], and peritoneum [17].

Endometriosis is a benign disease characterized by proliferation, infiltration, and malignant behaviors, where the ectopic endometrium repeatedly bleeds into the ovarian cortex and adheres to the surrounding ovarian epithelium to form a cyst. Implants gradually fill with a large number of decomposed blood cells, and excess iron from the anaerobic metabolism of red blood cells may result in toxicity, as iron overload has been linked to pathological disorders, which in turn may cause genetic mutations, hyperplasia, and even the increased malignant potential of the surrounding cells and tissues [18, 19]. However, the histological changes and molecular mechanism by which this process occurs remain elusive. In the present study, we first collected human atypical ovarian endometriosis tissues performed by two pathologists. Immunohistochemical analysis found that compared with normal ovarian endometriosis, the protein levels of GPR30 and the iron metabolite TfR in the human atypical ectopic endometrium were significantly increased. In addition, we also studied the relationship between GPR30 and Pi3K in malignant human ovarian endometriotic cysts, and the expression levels were still related. The significant association between GPR30 and the iron metabolites TfR/Pi3K suggests that GPR30 is closely related to the atypical hyperplasia of epithelial cells and the iron metabolism of endometriotic cysts and may be related to precancerous lesions or malignant transformation of the ovarian epithelium induced by persistent iron overload. Further particular roles of GPR30 in the atypical ovarian epithelium under long-term iron overload should be considered for the next step of research.

To further study and confirm the expression of GPR30 and iron metabolites in atypical ovarian endometriotic cysts, we established an ovarian model of sustained iron overload, and iron-induced histomorphology changes were observed in the ovarian epithelium. For the 20 ovary models, iron overload was identified by Prussian blue staining, epithelial hyperplasia was observed in 12 ovaries, and atypical papillary hyperplasia was observed in 8 ovary samples. We also characterized the difference in Pi3K staining between hyperplastic and atypical hyperplastic epithelium. We found that Pi3K was significantly expressed in all atypical hyperplasia specimens (*n* = 8) and in only 1 of the 10 hyperplasia specimens examined. The perspective that Pi3K is closely related to the occurrence and development of tumors indicates that persistent iron overload may lead to precancerous lesions or early malignant transformation of the ovarian epithelium.

In recent years, GPR30, the G protein-coupled estrogen receptor, has been shown to mediate both rapid and long-term transcriptional events against estrogen under certain circumstances. GPR30 has also been involved in estrogenic signaling in malignant cells, such as breast cancer and endometrial cancer [20, 21], suggesting that GPR30 may act at the crossroads between cancer cells and these important components of the tumor microenvironment. Endometriosis is an estrogen-dependent chronic inflammatory disease [22], and high estrogen levels lead to pathologic disorders, such as progressive proliferation, invasion, and even carcinogenesis. However, the exact status and role of the novel estrogen-responsive receptor *GPR30* gene has not been well studied or evaluated in endometriosis lesions, such as atypical changes. To achieve this objective, our previous study described the difference in GPR30 expression between benign and malignant ovarian endometriosis cysts. We found significant GPR30 expression in malignant ovarian endometriotic cysts compared to similar benign cysts, indicating that GPR30 may produce a wide range of responses to estrogen in endometriosis-associated ovarian carcinoma.

Additionally, in the present study, we further investigated a possible sequential progression from endometriosis through typical to atypical endometriosis by assessing *GPR30* at the gene level in iron-induced atypical ovarian epithelium. We found that the mRNA and protein expression levels of GPR30 were significantly higher in the atypical papillary hyperplasia group than in the typical hyperplasia and control groups. This finding suggests that there was transcriptional activation of *GPR30* gene expression in atypical hyperplasia, which led to a significant increase in the secreted expression of GPR30, as detected by western blotting, which is consistent with the changes in GPR30 expression levels in human atypical ovarian endometriosis tissues. Moreover, our results also demonstrated that GPR30 protein upregulation was significantly correlated with Pi3K in the iron-induced atypical ovarian epithelium. Increased Pi3K activity is associated with a variety of cancers and plays an extremely important role by mediating the carcinogenic processes of tumorigenesis, invasion, and metastasis, especially in the proliferation of normal epithelial cells to cancer cells [23]. In addition, studies have shown that the GPR30-Pi3K pathway is involved in cancer cell proliferation [24, 25].

Our previous study first hypothesized that GPR30 may be involved in the major pathologies of atypical changes in human ovarian endometriosis tissues and in the rat ovarian epithelium with persistent microinjection of iron. The significant association between GPR30 and the iron metabolites TfR/Pi3Ks suggests that GPR30 may be related to atypical hyperplasia of the ovarian epithelium under iron overload, which sheds light on the molecular mechanisms of precancerous transformation associated with ovarian endometriosis. However, the sample size should be expanded, and the relationship between GPR30 and iron metabolism in ovarian endometriosis should be further studied.

## Figures and Tables

**Table 1:** Analysis of IHC, HScore in human ovarian endometriotic cysts (mean ± SD).

	Positive rate		HScore	
	Atypical	Control	Atypical	Control
GPR30	20/20 (100%)	3/20 (15%)	305 (0-356)	62 (0-180)
TfR	20/20 (100%)	6/20 (30%)	276 (0-312)	75 (0-170)

HScore = *Σ*pi(*i* + 1): *i* is the intensity of staining, pi is the percentage of cells with positive staining/the total number of tested cells, and 1 is the correction factor. *P* < 0.05 *Atypical* vs. control.

**Table 2:** Pairwise correlation between the expression of GPR30, TfR, and Pi3K proteins.

GPR30	Pi3K		TfR		*P*
	Low^∗^	High^∗^	Low^∗^	High^∗^	
Low	1	2	0	0	0.001
High	3	14	1	19	

∗ Intensities of protein expressions in human ovarian atypical endometriotic cysts.

**Table 3:** Statistical analysis of GPR30 mRNA and protein.

Group	a	b	c	
mRNA	0.831 ± 0.003	0.132 ± 0.025	0.056 ± 0.012	*p*1
Protein	0.838 ± 0.001	0.127 ± 0.003	0.047 ± 0.003	*p*2

Group a: represents the atypical ovarian epithelium; group b: represents the hyperplasia ovarian epithelium; group c: control group. *p*1: the mRNA expression level of GPR30 in group a compared which in group b/c < 0.05; *p*2: the protein expression level of GPR30 in group a compared which in group b/c < 0.05.

**Table 4:** Analysis of IHC, HScore in different groups (mean ± SD).

	Positive rate		HScore		
	Atypical	Hyperplasia	Atypical	Hyperplasia	
Pi3K	8/8 (100%)	1/12 (8.3%)	323 (0-358)	12 (0-110)	*P* < 0.05
GPR30	8/8 (100%)	3/12 (25%)	309 (0-325)	25 (0-180)	*P* < 0.05

HScore = *Σ*pi(*i* + 1): *i* is the intensity of staining, pi is the percentage of cells with positive staining/the total number of tested cells, and 1 is the correction factor. *P* < 0.05 atypical vs. hyperplasia.

**Table 5:** Pairwise correlation between the expression of GPR30 and Pi3K proteins.

Pi3K			
GPR30	Low∗	High∗	*P*
Low	0	1		
High	0	7	0.001	

∗ Intensities of protein expressions in rat atypical ovarian epithelium.

## Data Availability

The underlying data supporting the results are available at the corresponding author.
